# Effectiveness of a Web-Based Self-Help Program for Suicidal Thinking in an Australian Community Sample: Randomized Controlled Trial

**DOI:** 10.2196/jmir.8595

**Published:** 2018-02-14

**Authors:** Bregje AJ van Spijker, Aliza Werner-Seidler, Philip J Batterham, Andrew Mackinnon, Alison L Calear, John A Gosling, Julia Reynolds, Ad JFM Kerkhof, Daniela Solomon, Fiona Shand, Helen Christensen

**Affiliations:** ^1^ Centre for Mental Health Research Australian National University Canberra Australia; ^2^ Black Dog Institute Department of Medicine University of New South Wales Randwick Australia; ^3^ EMGO Institute for Health Care Research VU University Amsterdam Amsterdam Netherlands; ^4^ Department of Clinical Neuro and Developmental Psychology VU University Amsterdam Amsterdam Netherlands

**Keywords:** psychosocial interventions, randomized controlled trial, suicide

## Abstract

**Background:**

Treatment for suicidality can be delivered online, but evidence for its effectiveness is needed.

**Objective:**

The goal of our study was to examine the effectiveness of an online self-help intervention for suicidal thinking compared to an attention-matched control program.

**Methods:**

A 2-arm randomized controlled trial was conducted with assessment at postintervention, 6, and, 12 months. Through media and community advertizing, 418 suicidal adults were recruited to an online portal and were delivered the intervention program (Living with Deadly Thoughts) or a control program (Living Well). The primary outcome was severity of suicidal thinking, assessed using the Columbia Suicide Severity Rating Scale.

**Results:**

Intention-to-treat analyses showed significant reductions in the severity of suicidal thinking at postintervention, 6, and 12 months. However, no overall group differences were found.

**Conclusions:**

Living with Deadly Thoughts was of no greater effectiveness than the control group. Further investigation into the conditions under which this program may be beneficial is now needed. Limitations of this trial include it being underpowered given the effect size ultimately observed, a high attrition rate, and the inability of determining suicide deaths or of verifying self-reported suicide attempts.

**Trial Registration:**

Australian New Zealand Clinical Trials Registry ACTRN12613000410752; https://www.anzctr.org.au/ Trial/Registration/TrialReview.aspx?id=364016 (Archived by WebCite at http://www.webcitation.org/6vK5FvQXy); Universal Trial Number U1111-1141-6595

## Introduction

Suicidal thinking is common and often precedes suicidal plans and attempts [1,2]. Effective treatments exist for suicidal thinking [3,4], but many individuals do not seek help due to factors such as low suicide literacy (ie, having little knowledge about suicidality), lack of perceived need, preference to manage the problem alone, stigma, shame, beliefs about receiving professional help, fear of hospitalization, structural factors (eg, time and finances), and having previously experienced negative encounters with health care professionals [5-9]. Providing anonymous self-help online may address these barriers to help-seeking. The internet provides an avenue to reach people with suicidal thinking and offers the potential to prevent escalation to suicidal behavior or suicide itself. While there is good evidence that Web-based programs are effective for a variety of mental health problems [10,11], suicidality is often an exclusion criteria in these trials and interventions [12].

A Dutch trial of a self-guided online intervention for suicidal thinking reported significant reductions in suicidal thoughts relative to the waitlist control condition that included access to a website with psychoeducational material about suicide [13,14]. These results require replication and extension. In the Dutch trial, respondents with a high depression and/or suicidality score were excluded, participants could not maintain anonymity as they had to provide their own and their general practitioner contact details, and follow-up was limited to 3 months. This trial evaluated an English language version of the Dutch self-help intervention, used a more broadly recruited community-based sample, did not exclude those with severe depressive symptoms, permitted anonymity, followed up over 12 months, and compared the intervention to an attention-matched control program to ensure an equal amount of material was presented to each group. It was hypothesized that, compared to controls, participants randomized to the active intervention would experience reduced severity of suicidal thinking at postintervention and 6- and 12-month follow-up. Improvements on secondary outcomes (suicidal ideation, suicidal behavior, reasons for living, perceived burdensomeness, thwarted belongingness, acquired capability, depression, hopelessness, anxiety, panic, rumination, alcohol use, insomnia, physical health, mental health, and physical functioning) were also expected. We sought to identify any potential moderating effects of sex, age, depression severity, history of attempted suicide, chronicity of suicide risk, and the effect of adherence on outcomes.

## Methods

### Trial Design

The study was a 2-arm randomized controlled trial delivered entirely online consisting of a treatment condition, Living with Deadly Thoughts (LwDT), and an attention-matched control condition, Living Well. There were 4 measurement occasions: baseline, postintervention (6 weeks after baseline) and 6- and 12-month follow-up. Full details on the study methodology can be found in the trial protocol [15]. The trial is reported in accordance with the CONSORT-EHEALTH checklist (see Multimedia Appendix 1).

### Participants

Participants were recruited between November 2013 and December 2015 through online media forums including websites, social networking websites, and advertizing on search engines. A link to a welcome screen included an invitation to provide consent and complete the online screening procedure.

Eligibility criteria were as follows: aged 18 to 65 years, valid email address, access to a reliable internet connection, located in Australia, fluent in English, no history of a diagnosed psychotic disorder, currently experiencing suicidal thoughts, and no suicide attempts in the past month. These were assessed using single, self-report questions. Unlike the original trial [13], no restrictions were placed on the severity of suicidal thinking or depression.

Respondents who did not meet inclusion criteria were redirected to a “thank you” page listing referral information. Respondents who were excluded based on a recent suicide attempt were also provided with the opportunity to submit their phone number to receive a phone call from the Suicide Call Back Service (SCBS), a 24/7, Australia-wide, not-for-profit service that provides telephone counseling (www.suicidecallbackservice.org.au). Participants were informed that the study was not intended to replace treatment as usual and were encouraged to seek or continue other treatment.

### Interventions

#### Active Condition: Living With Deadly Thoughts

LwDT is an adapted but closely aligned translation of the Dutch Web-based program *Leven onder Controle* (literally “Living under Control”). The content is drawn from principles of cognitive behavior therapy [16] and dialectical behavior therapy [17], and the program’s goal is to reduce the severity of suicidal thinking.

The program consists of 6 online modules. Participants are instructed to complete 1 module per week and to spend 30 minutes per day using the program. Each module contains 4 components: (1) theory, (2) a weekly assignment, (3) 2 to 3 exercises, and (4) optional exercises to help consolidate relevant information and skills. The modules become available in a fixed sequence, 4 days after the start of the previous module, regardless of completion, and remain available throughout the intervention period. Access to referral information via a “get help now” link is available on every Web page. A safety procedure included monitoring and intervention (if required) by the SCBS (see Safety Procedures).

#### Attention Control Condition: Living Well

The attention control condition was matched to the active program in length, style, and availability. It involved a 6-week modular online learning course containing lifestyle information on (1) nutrition, (2) a healthy home environment, (3) a healthy weight, (4) a healthy heart, (5) healthy skin, and (6) a healthy mouth. Participants in the control condition received the safety protocol procedure, including monitoring and intervention (if required) by the SCBS (see Safety Procedures).

### Primary Outcome Measure: Severity of Suicidal Thinking

The primary outcome was severity of suicidal thinking, assessed using the Intensity of Suicidal Ideation subscale of the Columbia Suicide Severity Rating Scale (C-SSRS) [18]. This subscale comprises 5 items, each rated for frequency, duration, controllability, deterrents, and reasons for ideation.

### Secondary Outcome Measures

Secondary outcome measures included presence of ideation and behavior, measured by the Suicidal Ideation and Suicidal Behavior subscales of the C-SSRS, respectively; reasons for living, using the Brief Reasons for Living Scale [19]; perceived burdensomeness and thwarted belongingness, assessed by the Interpersonal Needs Questionnaire [20]; acquired capability, measured by Acquired Capability for Suicide Scale [20]; depression, measured by Centre for Epidemiological Studies Depression Scale [21]; hopelessness, assessed by the Burns Hopelessness Scale (D Burns, personal communication); anxiety, measured by the 7-item Generalized Anxiety Disorder scale [22]; panic, using the Panic Syndrome subscale of the Brief Patient Health Questionnaire [23]; rumination, assessed by the Ruminative Response Scale [24]; alcohol consumption, using the Alcohol Use Disorders Identification Test–Consumption [25]; and insomnia, assessed by the Insomnia Severity Index [26]. Health-related quality of life was measured using the Short Form–12 [27], and health and disability were measured by the World Health Organization Disability Assessment Schedule [28]. The Suicidal Ideation Attributes Scale (SIDAS) [29] was included to validate its psychometric features.

Standard demographic information including sex, age, relationship status, education, and employment status were collected together with self-reported lifetime suicide attempts and adherence to program by website usage.

### Safety Procedures

Given the vulnerable, at-risk population that this study targeted, safety procedures were designed in collaboration with the SCBS with the goal of protecting and assisting participants while enabling them to maintain anonymity within the trial and considering ethics and clinical obligations. The ability to remain autonomous in the decision to make contact with services as much as possible was also important, as this is consistent with the concept of self-help and patient empowerment.

The safety procedures required eligible participants to make contact with the SCBS during enrollment to obtain a unique identification code, so that codes rather than names could be used in all subsequent communication between the research team and SCBS. On each measurement occasion, the first 3 items of the intensity of suicidal ideation section of the C-SSRS were used to detect high risk. Scores above a specified cutoff (a score of 5 on any of these items) alerted the participant to contact the SCBS. Not doing this within 2 days triggered a reminder email to the participant, as well as a message to the SCBS asking them to contact the participant (if contact information was available, otherwise no further action was taken). The provision of contact information to the research team or to SCBS was a voluntary option at enrollment for the trial.

Conversations between participants and SCBS staff (counselors, social workers, and psychologists) were conducted according to SCBS protocols without input from the research team.

### Sample Size

Power to detect change in suicidal thoughts on the C-SSRS was based on an effect size of 0.3 (Cohen *d*), as found in the original trial [13]. To detect this effect size with 80% power and alpha=.05, assuming *r*=.5 between the baseline and postintervention measures and allowing for up to 30% attrition (again informed by the previous trial), 285 participants per arm would be required to yield at least 200 completers. Therefore, the aim was to recruit 570 participants into the trial.

### Randomization and Sequence Generation

Randomization to the active or control condition occurred on a 1:1 ratio using a block design (4 participants per block), stratified by sex and severity of suicidal thinking (high severity was defined as endorsing yes on the fifth item of the Suicidal Ideation subscale of the C-SSRS, which assesses active suicidal ideation, defined as having a specific plan and intent). The randomization procedure was incorporated into the website and was fully automated.

### Blinding

No research personnel were involved in the delivery of the interventions. Participants were not informed whether their assigned condition would receive the active or control program. However, it is likely that participants were able to discern their allocation based on the nature of the intervention they received, which increases risk of detection bias. All outcome measures were self-report and completed via an online portal. All research personnel (except those involved with the day-to-day management of the trial), remained blind to intervention allocation.

### Procedure

Ethical approval was obtained from the human research ethics committees of the University of New South Wales (HC13117) and the Australian National University (2012/471). The trial was registered at Australia New Zealand Clinical Trials (ACTRN12613000410752) and has a Universal Trial Number (U1111-1141-6595).

Following the screening process, eligible participants received instructions specifying that participation required making contact with the SCBS to obtain a unique identification code. Respondents provided consent, a valid email address, a name/nickname, and a telephone number (nonmandatory). Participants were then invited to complete baseline measures after which they were randomized.

Six weeks after randomization (after completing the program), participants were invited to complete the postintervention questionnaire. Follow-up assessments took place at 6- and 12-month follow-up. Participants who stopped using the program but who did not formally withdraw continued to be emailed follow-up questionnaires. Participants in the control group were given access to LwDT after the 12-month follow-up assessment.

### Statistical Analysis

Primary analyses were performed on an intention-to-treat basis. All outcomes were tested using planned contrasts of mean change scores from pre- to postintervention via a mixed-model repeated measures (MMRM) analytic approach. An unstructured variance-covariance matrix was used to model within-individual dependencies. MMRM analysis uses all available data and yields estimates of effect under restricted maximum likelihood. The test of the efficacy of the intervention was based on the interaction between intervention condition and time. Supplementary analyses on the primary outcome were conducted for individuals who completed more than half of the program, as defined by completion of 4 or more of the available 6 modules. Using the whole sample, moderation of the effectiveness of the intervention by sex, age, baseline depression severity, history of suicide attempts (nil vs at least one), and chronicity of suicidal thinking (total number of months spent thinking about suicide) was examined. Moderation was tested using an identical MMRM approach with the addition of the moderating variable of interest as a factor into the model with 2- and 3-way interactions with group and time [30]. All analyses were performed using SPSS version 24 (IBM Corp).

## Results

### Participant Characteristics

**Figure 1:**
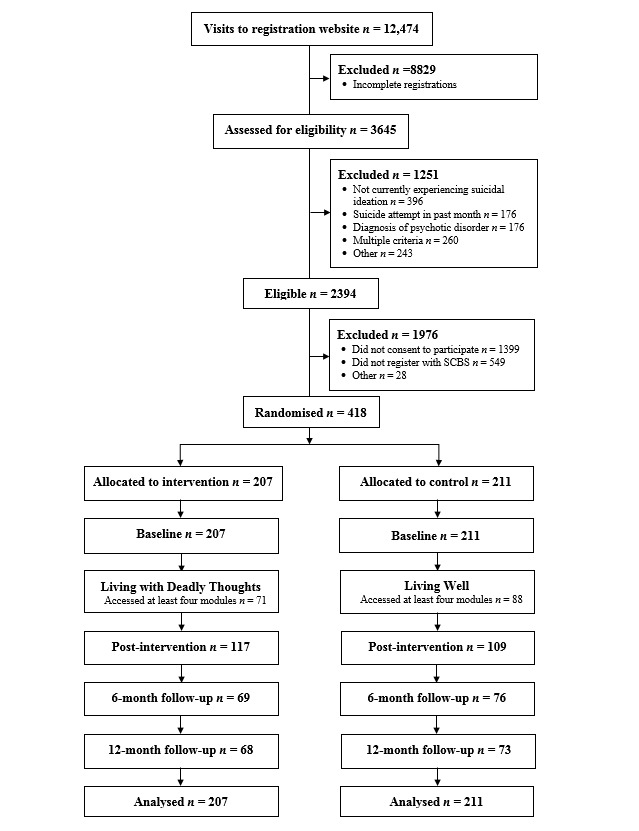
Participant flow diagram.

Participant flow is shown in Figure 1. The registration Web page received 12,474 visits during recruitment. Almost three-quarters (8829/12,474, 70.78%) of these visits were by respondents who failed to complete the screening questionnaire. Two-thirds (2394/3645, 65.68%) of those who completed screening were eligible to participate. Of these, 41.56% (995/2394) consented to participate but fewer than half (446/995, 44.8%) contacted the SCBS to obtain an identification code. Almost all (418/446, 93.7%) of those who obtained an identification code completed the baseline assessments and were randomized.

Sample characteristics are provided in Table 1. At baseline, the majority of participants were female (323/418, 77.3%), lived in a metropolitan area (253/418, 60.5%), and had completed secondary-school level education (316/418, 75.6%). A considerable proportion were married or in a de facto relationship (160/418, 38.3%). Mean age of the total sample was 40.6 (SD  11.9) years and the majority were employed (248/418, 59.3%). There were no differences at baseline between the 2 conditions in demographic characteristics (all *P* values were greater than .05).

On average, the severity of suicidal thinking fell in the very high range, with one-third of the sample reporting multiple past suicide attempts. Symptoms of psychopathology were elevated, including depression, hopelessness, anxiety, panic, and sleep disturbance. Compared to normative samples, health-related quality of life was lower and impairment was higher. There were no group differences on baseline clinical variables, with the exception of the intensity of suicidal thinking, which was higher in the intervention group relative to the control group, *t*_416_=2.71, *P*=.007.

### Attrition

Attrition was 45.9% (192/418) at postintervention, 65.3% (273/418) at 6 months, and 66.3% (277/418) at 12-month follow-up (see Figure 1). Chi-square tests showed that individuals who dropped out were similarly distributed across the control and intervention groups (postintervention, χ^2^_1_=0.33, *P*=.57; 6 months, χ^2^_1_=0.33, *P*=.56; 12 months, χ^2^_1_=0.14, *P*=.71). Baseline characteristics of those who dropped out indicated that they were more depressed, *F*_1,416_=8.42, *P*=.004; more anxious, *F*_1,416_=6.53, *P*=.01; reported greater levels of thwarted belongingness, *F*_1,416_=6.98, *P*=.009; increased frequency of suicidal behaviors, *F*_1,416_=6.42, *P*=.01; and higher levels of disability, *F*_1,416_=6.39, *P*=.01.

In terms of adherence to the program, there were no significant between-group differences in the number of modules completed, χ^2^_6_=9.26, *P*=.16. In the active intervention group, 8.2% (17/207) participants did not start the intervention (ie, did not access the first module), 91.8% (190/207) accessed at least 2 modules, 44.4% (92/207) accessed at least 3 modules, and 34.8% (72/207) accessed 4 or more modules. There were no significant between-group differences in baseline characteristics (all *P* values greater than .05), nor were there significant between-group differences in care received in a hospital setting, χ^2^_2_=4.19, *P*=.12, or from a general practitioner, χ^2^_2_=5.10, *P*=.08.

### Safety Procedures

Participants who scored above the cutoff on the C-SSRS at any time during the study were telephoned by the suicide crisis help-line. More participants in the intervention condition (59/207) were called compared to those in the control condition (37/211), χ^2^_1_=7.10, *P*=.008. Differences between groups in number of alerts per participant did not entirely explain overrepresentation of participants with alerts in the intervention group: 10 intervention versus 4 control participants had 3 or more alerts (Fisher exact test, *P*=.109). The breakdown of number of alerts per group is provided in Table 2.

Based on self-report data from the C-SSRS Suicidal Behavior subscale, 23 participants made a suicide attempt during the course of the study, with no differences between groups, χ^2^_1_=.03, *P*=.87 (11 vs 12 attempts, respectively, for control and LwDT).

### Intervention Effects on Primary Outcome: Severity of Suicidal Thinking

There was no overall group difference between the LwDT group and the control group on the primary outcome, severity of suicidal thinking at postintervention, *t*_245.51_=−1.20 (95% CI −1.84 to −0.44), *P*=.23; 6 months, *t*_172.57_=−0.88 (95% CI −2.17 to −0.84), *P*=.38; and 12 months, *t*_163.89_=−3.58 (95% CI −1.75 to −1.22), *P*=.72.

**Table 1:** Sample characteristics of participants in the intervention (Living with Deadly Thoughts) and control (Living Well) groups.

Characteristic		Intervention (n=207)	Control (n=211)	*P* value
Gender (female), n (%)		160 (77.3)	163 (77.3)	.37
Age, years, mean (SD)		39.53 (11.94)	41.73 (11.86)	.06
Educational history (completed secondary school), n (%)		158 (76.3)	158 (74.9)	.42
Marital status (married or de facto), n (%)		87 (42.0)	73 (34.6)	.27
Employment (employed), n (%)		121 (58.5)	127 (60.2)	.47
**Area of residence, n (%)**				
	Metropolitan	127 (61.4)	126 (59.7)	.95
	Regional, rural, or remote	79 (38.2)	84 (39.8)	.63
**Lifetime history of suicide attempts, n (%)**				
	Never	90 (43.5)	101 (47.9)	.37
	Once	46 (22.2)	43 (20.4)	.65
	More than once	71 (34.3)	67 (31.8)	.58
**Adherence to program, n (%)**				
	Accessed at least 4 modules	71 (34.3)	88 (41.7)	.14

**Table 2:** Alerts per group.

Number of alerts	Intervention (n=207)	Control (n=211)
1	34	25
2	15	8
3	7	1
4	2	2
5	1	1

However, both groups showed significant reductions in the severity of suicidal thinking relative to baseline at postintervention, *t*_244.85_=−4.55, *P*<.001 (95% CI −2.64 to −1.05), *d*=0.56; 6-month follow-up, *t*_171.78_=−5.46, *P*<.001 (95% CI −3.9 to −1.83), *d*=0.72; and 12-month follow-up, *t*_163.93_=−6.72, *P*<.001 (95% CI −4.55 to −2.48), *d*=0.77.

Participants who completed at least 4 of the 6 modules in the LwDT condition were compared to those who completed at least 4 modules of the control program. Analyses indicated that completers of the LwDT program experienced a reduction in severity of suicidal thinking relative to completers of the control program at posttest, *t*_150.45_=−2.16, *P*=.033 (95% CI –2.95 to –0.12), *d=* 0.60, but not at 6-month, *t*_117.62_=−1.40, *P*=.17, or 12-month follow-up, *t*_108.02_=.57, *P*=.57.

### Moderation of Intervention Effects on Severity of Suicidal Thinking

Moderation of the effectiveness of the intervention by sex, age, baseline depressive symptoms, history of attempted suicide, and chronicity of suicidal thinking was investigated by examining 3-way interactions between group, time, condition, and the moderating variable of interest. Severity of suicidal thinking was not moderated by sex, *F*_3,178.36_=1.56, *P*=.20; age, *F*_3,177.43_=0.43, *P*=.73; baseline depression symptoms, *F*_3,192.9_=0.52, *P*=.70; or history of previous attempts, *F*_3,182.21_=0.31, *P*=.82. However, a moderation effect on the primary outcome was detected for chronicity of suicidal thinking, *F*_3,187.37_=3.13, *P*=.03, with evidence that the LwDT program was more effective at reducing the severity of suicidal thinking in participants who had spent less time (fewer months) thinking about suicide.

### Intervention Effects on Secondary Outcomes

The LwDT program, compared with the LW program, had no significant effect on secondary outcomes except for anxiety (see Multimedia Appendix 2), which showed a greater decrease at 12 months (*t*_185.55_=−2.04, *P*=.04). This effect was not significant at posttest (*P*=.65) or 6 months (*P*=.17).

Across both groups (LwDT and Living Well) there was a significant reduction at all time points in the following: suicidal ideation (as measured by the C-SSRS and by the SIDAS), suicidal behavior, burdensomeness, depression, hopelessness, anxiety, panic, physical health, mental health, and physical functioning. There were also significant decreases in both groups for thwarted belongingness at 6- and 12-month follow-up, rumination at 6- and 12-month follow-up, and sleep difficulties at posttest and 12-month follow-up. Test statistics for these within-group comparisons are available from the authors upon request.

Variables that did not significantly improve over time were acquired capability for suicide, reasons for living, and alcohol use, although a group difference emerged at 12-month follow-up, with those in the intervention group having lower alcohol use scores than those in the control group, *t*_145.59_=–2.152, *P=*.03.

## Discussion

### Principal Findings

Regardless of intervention allocation, participants’ level of suicidal thinking reduced over time, with no difference between the groups at postintervention or 6- or 12-month follow-up. Also, there was no evidence of between-group differences on most of the secondary variables.

These findings are discrepant from the original study of LwDT, where a significant difference was found for the intervention compared to a waitlist control group in suicidal thoughts and worry [13]. Several differences between these studies may account for this. First, the sample in the current trial was more severe than those in the original trial, both in terms of suicidality and depression. If the same criteria had been used in the original trial, 50% of those entering this trial would have been excluded. The findings from the moderation analysis might be interpreted to suggest that the intervention is indeed more suitable for those with less chronic suicidal thinking, as these participants had better outcomes. Severe levels of suicidality and depression are associated with poor motivation and impaired cognitive functioning, including poor concentration and attention [31]. However, baseline level of depression did not impact the effectiveness of the intervention, although the possibility remains that suicidal severity could interfere with program completion. It should also be noted here that the Dutch trial found more pronounced effects for severe suicidality [13,32], which contradicts the current findings. Another possibility relates to help-negation, which is the decreased propensity to engage in help-seeking behaviors as a consequence of more severe levels of suicidal ideation. Individuals who dropped out of the study after baseline had more severe levels of depression, anxiety, and suicidal behaviors, a finding that indicates that severe symptoms may interfere with completion of the program and that LwDT may be better suited to individuals with moderate symptom levels and less severe suicidality.

A second difference is that the original study involved a waitlist control condition, while the present trial had an attention-control condition. A number of features of the control intervention, including the fortnightly monitoring during the active phase, risk-triggered alerts, proactive follow-ups (if required) by the SCBS, and delivery of helpful lifestyle materials may have contributed to the reduction in suicidality, in addition to an expected standard placebo effect. The proactive nature of the intervention in the control group may have contributed to the improvement over time, obscuring any addition impact of the LwDT intervention. The monitoring of suicide ideation itself combined with the intervention by the call back service provides a safety protocol procedure that is known to have an effect [33].

A key procedural aim of this study was to provide LwDT without requiring participants to register with their doctors. Lack of anonymity poses an obstacle to help-seeking for many individuals [34]. Although the involvement of SCBS provided a solution to the issue of anonymity, it unfortunately created another challenge. Some participants attempted to call and, as is the case with many crisis services, were placed on hold and unable to register for the trial immediately. Some participants provided anecdotal reports of attempting to get through several times before giving up. Others indicated that they did not wish to contact SCBS for a range of reasons, including previous negative experiences with crisis support helpline services. Of the participants who provided consent, 55% did not go on to register with SCBS and were therefore unable to take part in the trial. LwDT, if delivered under real-world conditions outside the context of a research trial, would not require compulsory contact with a crisis support service such as SCBS. In the current context, it is a limitation that created a barrier to participation and may impact the generalizability of results.

A related issue was attrition and the role that general practitioners may have played in the original trial. In this study, attrition was 46% at postintervention, which is at the higher end of rates of attrition of online interventions [35,36]. However, these rates refer to depression and anxiety online treatment programs and not programs specifically for suicidality. For online treatments, predictors of dropout include severity and chronicity [35]. Although speculative, it is likely that the severely suicidal nature of the sample in this trial may have contributed to the relatively high level of dropout. The dropout rate in this study was also higher than that in the original trial (11%), which most likely arose as a product of 2 factors: the monitoring role played by the participants’ regular doctor and the exclusion of severely suicidal and depressed participants. Some online programs that are completed in combination with professional support report lower rates of attrition [37].

The difference in number of alerts in this trial between the groups was also notable, with significantly more alerts triggered in the intervention group. This difference was not observed in the Dutch trial. Reasons for this difference are speculative but may be due to an imbalance of high-need people between the groups (as intensity of suicidal ideation was higher at baseline in the intervention group). Alternatively, the high alert rate may indicate that the intervention has an effect on help-seeking from a telephone helpline—something that is actively encouraged in the LwDT program. As registration with a helpline was mandatory for this trial, this may have lowered the threshold for calling, a factor that did not apply in the Dutch trial.

### Limitations

The current trial had a number of other limitations. First, the target sample size for the trial was not met due to time constraints. The study was therefore underpowered and, given the high attrition rate, any smaller differences would not have been detected. Determination of any deaths by suicide was not possible within the context of the trial, given the lack of access to official records and anonymity of the participants. While this trial was not powered to detect group-level differences in attempted suicides or suicide deaths, future trials in this field recruiting larger numbers of participants would do well to incorporate an analysis of patient records to assess deaths by suicide over an extended period. Further, the incidence of suicide attempts could not be verified given the reliance on self-report rather than hospital and medical records.

### Conclusion

The results of this study indicate that completing an online intervention is associated with reduced suicidal thinking and psychological symptoms over time, but this may be due to the exposure to a structured program, monitoring, and safety procedures. Further assessment with a waitlist control group would be required to confirm any advantage of the structured program above that of the safety procedures, and such a trial is unlikely to receive ethical approval.

This study has several clinical and research implications. Overall, there was no evidence that the LwDT program is harmful and, looking to the original Dutch trial, may be beneficial to those who have a less chronic history of suicidal thinking. This is consistent with clinical guidelines for the use of low-intensity interventions (ie, National Institute for Health and Care Excellence guidelines) for individuals who have low to moderate symptom levels. However, with the Dutch trial indicating more pronounced effects for people with a history of suicide attempt and this trial showing less effectiveness for those with a longer history of suicidal thinking, future research should investigate what target group might benefit most from online self-help, what adjustments might need to be made to the program to accommodate for various degrees of suicidality, and how the delivery of the program might improve outcomes at both ends of the spectrum. For example, incorporation in a stepped care model or tailoring of the program to individual needs might increase effectiveness across levels of severity.

Furthermore, the results that were achieved are of interest because they suggest that interventions are associated with a drop in symptoms. Research in support of crisis lines is also built on this type of outcome study and used to support their effectiveness. Also notable, with respect to helplines, is that even though it would make sense to implement programs such as LwDT in organizations that provide crisis support, less than half of eligible respondents enrolled with the SCBS, indicating that this could also be a deterrent. This should be taken into account when preparing such programs for use outside of a research context. Finally, the type of high-quality trial methodology—a randomized controlled trial—such as ours may not be the best methodology to observe outcomes, given the constraints outlined above.

We have shown that the provision of a self -help program via the internet is feasible but that further research is needed to better understand the types of interventions, both online and offline, that could assist people experiencing suicide ideation and the most useful settings in which to situate these interventions.

## Appendix

Multimedia Appendix 1 CONSORT‐EHEALTH checklist (V 1.6.1).

Multimedia Appendix 2 Estimated marginal means from mixed-model repeated measures for primary and secondary outcomes at each time point for the intervention condition.

## Abbreviations

C-SSRS: Columbia Suicide Severity Rating Scale
LwDT: Living with Deadly Thoughts
MMRM: mixed-model repeated measures
NHMRC: National Health and Medical Research Council
SCBS: Suicide Call Back Service
SIDAS: Suicidal Ideation Attributes Scale
