# Supplementary material for: Effectiveness of a Web-Based Self-Help Program for Suicidal Thinking in an Australian Community Sample: Randomized Controlled Trial
Source: J Med Internet Res. 2018 Feb 14;20(2):e15. doi: 10.2196/jmir.8595 (PMC5830610; doi:10.2196/jmir.8595)
Supplement: Multimedia Appendix 2 [file jmir_v20i2e15_app2.pdf]

Multimedia Appendix 2. Estimated marginal means (s.e.) from MMRM for primary and secondary outcomes at each time point for the intervention condition (LwDT) estimated from mixed-model repeated measures analyses.

	Living with Deadly Thoughts (intervention)				Living Well (control)				Condition × time effect		
	**Baseline**	**Post-intervention**	**6 months**	**12 months**	**Baseline**	**Post-intervention**	**6 months**	**12 months**			
	***n* = 207**	***n* = 109**	***n* = 69**	***n* = 68**	***n* = 211**	***n* = 117**	***n* = 76**	***n* = 73**	***F***	***df***	***p***
**Primary outcome**											
Severity (C-SSRS)	15.68 (0.21)	13.14 (0.42)	12.14 (0.56)	12.43 (0.55)	14.85 (0.22)	13.00 (0.41)	11.98 (0.53)	11.33 (0.53)	0.89	3, 182.56	0.445
**Secondary outcomes**											
**Suicidality**											
Ideation (C-SSRS)	3.11 (0.08)	2.43 (0.15)	2.02 (0.18)	2.06 (0.19)	3.00 (0.08)	2.16 (0.15)	1.99 (0.18)	1.69 (0.19)	0.75	3, 182.81	0.523
Behaviour (C-SSRS)	1.42 (0.12)	0.87 (0.13)	1.10 (0.15)	0.87 (0.17)	1.43 (0.12)	1.02 (0.13)	1.04 (0.14)	1.07 (0.16)	0.59	3, 191.69	0.622
Ideation (SIDAS)	28.03 (0.71)	21.48 (1.04)	17.41 (1.53)	16.38 (1.39)	25.37 (0.70)	20.41 (1.02)	18.88 (1.48)	15.99 (1.36)	1.44	3, 174.14	0.232
Reasons for living (BRFL)	37.8 (0.79)	38.01 (1.02)	39.28 (1.23)	37.66 (1.20)	37.42 (0.78)	37.01 (1.00)	36.86 (1.18)	36.17 (1.17)	0.70	3, 181.00	0.553
Burdensomeness (INQ)	25.69 (0.68)	22.93 (0.84)	21.07 (1.10)	19.26 (1.02)	24.56 (0.68)	21.23 (0.82)	20.12 (1.05)	19.84 (0.99)	0.95	3, 175.58	0.418
Belongingness (INQ)	28.06 (0.50)	25.79 (0.72)	24.46 (1.00)	24.83 (0.95)	28.26 (0.49)	27.08 (0.70)	24.85 (0.95)	24.53 (0.92)	0.63	3, 184.53	0.596
Capability (ACSS)	10.63 (0.31)	11.01 (0.40)	11.06 (0.44)	10.43 (0.46)	10.79 (0.31)	10.82 (0.40)	11.14 (0.43)	10.92 (0.45)	0.49	3, 172.01	0.689
**Symptomatology**											
Depression (CES-D)	40.82 (0.67)	33.05 (1.12)	31.22 (1.49)	30.9 (1.40)	39.72 (0.66)	32.58 (1.09)	29.35 (1.41)	31.68 (1.36)	0.63	3, 188.13	0.594
Hopelessness (BHS)	13.11 (0.34)	11.26 (0.51)	9.90 (0.69)	9.40 (0.65)	12.54 (0.33)	9.85 (0.50)	9.20 (0.65)	9.24 (0.63)	0.61	3, 188.75	0.609
Anxiety (GAD-7)	13.40 (0.35)	11.73 (0.50)	11.65 (0.59)	9.88 (0.59)	13.14 (0.35)	11.17 (0.49)	10.23 (0.56)	11.29 (0.57)	3.95	3, 191.75	**0.009**
Panic (PHQ-Panic)	3.03 (0.15)	2.70 (0.20)	2.87 (0.24)	2.56 (0.25)	3.10 (0.15)	2.68 (0.20)	2.20 (0.23)	2.39 (0.24)	1.75	3, 193.00	0.159
Rumination (RRS)	27.21 (0.42)	26.48 (0.54)	25.75 (0.65)	25.9 (0.63)	26.60 (0.42)	26.00 (0.53)	24.52 (0.62)	24.10 (0.61)	0.81	3, 183.64	0.491
Alcohol (AUDIT-C)	3.13 (0.21)	2.88 (0.22)	3.11 (0.24)	2.71 (0.27)	3.02 (0.20)	3.06 (0.22)	3.20 (0.23)	3.27 (0.26)	1.78	3, 162.25	0.153
Sleep (ISI)	15.44 (0.41)	13.60 (0.58)	13.46 (0.71)	12.11 (0.68)	15.26 (0.41)	14.16 (0.57)	13.96 (0.68)	13.37 (0.66)	0.95	3, 183.79	0.418
**Quality of life**											
Physical health (SF-12-PHS)	40.14 (0.45)	19.31 (0.61)	52.28 (0.52)	52.12 (0.54)	41.19 (0.45)	40.14 (0.45)	51.91 (0.52)	51.88 (0.54)	0.90	3, 416.00	0.443
Mental health (SF-12-MHS)	35.89 (0.41)	49.40 (0.85)	53.58 (0.79)	53.93 (0.76)	36.21 (0.41)	49.70 (0.84)	52.97 (0.79)	53.67 (0.75)	0.29	3, 416.00	0.835
Physical functioning (WHODAS)	40.98 (1.29)	33.95 (1.76)	34.55 (2.21)	31.2 (2.05)	39.05 (1.28)	34.13 (1.72)	33.13 (2.17)	32.92 (2.00)	0.99	3, 171.73	0.399

All values are estimated marginal mean (s.e.), from mixed-model repeated measures analyses. ACSS, Acquired Capability for Suicide Scale; AUDIT-C = Alcohol Use Disorders Identification Test Consumption; BHS, Burns Hopelessness Scale; BRFL, Brief Reasons for Living Scale; C-SSRS, Columbia Suicide Severity Rating Scale; CES-D, Center for Epidemiologic Studies Depression Scale; GAD-7 = 7-item Generalized Anxiety Disorder scale; INQ, Interpersonal Needs Questionnaire; ISI, Insomnia Severity Index; PHQ-Panic, panic syndrome subscale of the Brief Patient Health Questionnaire; RRS, Rumination Response Scale; SF12-MCS, Short Form-12 Mental Health Composite Scale; SF-12-PHS, Short Form-12 Physical Health Composite Scale; SIDAS, Suicidal Ideation Attributes Scale; WHODAS, World Health Organization Disability Assessment Schedule.
